# Integrating Syndromic Molecular Assays into Routine Diagnostic Microbiology: Benefits and Challenges

**DOI:** 10.3390/antibiotics15020182

**Published:** 2026-02-07

**Authors:** Sara Comini, Anna Maria Priori, Francesco Coppari, Matteo Sabbatini, Concetta Bruno, Matteo Boattini, Gabriele Bianco, Francesca Brecciaroli

**Affiliations:** 1 Operative Unit of Clinical Pathology, Carlo Urbani Hospital, 60035 Ancona, Italy; annamaria.priori@sanita.marche.it (A.M.P.); fracoppa00@gmail.com (F.C.); matteo.sabbatini@sanita.marche.it (M.S.); concetta.bruno@sanita.marche.it (C.B.); francesca.brecciaroli@sanita.marche.it (F.B.); 2 Microbiology and Virology Unit, University Hospital Città della Salute e della Scienza di Torino, 10100 Turin, Italy; matteoboattini@gmail.com; 3 Department of Public Health and Paediatrics, University of Torino, 10126 Turin, Italy; 4 Lisbon Academic Medical Centre, 1000-001 Lisbon, Portugal; 5 Department of Experimental Medicine, University of Salento, 73100 Lecce, Italy; gabriele.bianco@unisalento.it; 6 Microbiology and Virology Unit, Vito Fazzi Hospital, 73100 Lecce, Italy

**Keywords:** syndromic molecular diagnostics, FilmArray^®^, antimicrobial resistance, rapid diagnosis, bloodstream infections, antimicrobial stewardship

## Abstract

**Background/Objectives**: Rapid pathogen identification is essential to optimize antimicrobial therapy and improve patient outcomes, particularly in severe infections. Syndromic molecular diagnostics have been introduced to overcome the limitations of conventional culture-based methods. This study evaluated the diagnostic performance and real-life implementation of BioFire^®^ FilmArray^®^ syndromic panels compared with routine microbiological diagnostics. **Methods**: A total of 955 clinical specimens collected between 2022 and June 2025 were retrospectively analyzed, including positive blood cultures (*n* = 400), lower respiratory tract samples (*n* = 309), cerebrospinal fluid (*n* = 158) and stool specimens (*n* = 88). FilmArray^®^ BCID2, Pneumonia Plus, Meningitis/Encephalitis and Gastrointestinal panels were performed on the Biofire Fimarray^®^ instrument according to clinical indication and compared with conventional culture-based identification and phenotypic antimicrobial susceptibility testing. **Results**: Overall diagnostic concordance between BioFire^®^ FilmArray^®^ syndromic panels and conventional methods was high across all specimen types, with the highest positive percent agreement (PPA) observed for bloodstream infections (97.7%) and gastrointestinal pathogens (100%). In respiratory samples, the Pneumonia Plus panel detected a considerable number of microorganisms that could not be identified by culture, including viral pathogens and fastidious bacteria. Molecular detection of antimicrobial resistance markers showed excellent concordance with phenotypic profiles, with 100% agreement for CTX-M, carbapenemases (KPC, NDM, OXA-48-like, IMP), and vanA/B, while lower concordance was observed for *mecA/C* in staphylococci. In parallel, semi-quantitative bacterial loads provided by the Pneumonia Plus panel showed a strong essential agreement with culture-based quantification (97.4%, ±1 log_10_). Across all panels, syndromic testing significantly reduced diagnostic turnaround time. **Conclusions**: Syndromic molecular panels provide rapid and reliable simultaneous detection of pathogens, as well as early resistance marker detection, thereby supporting timely antimicrobial optimization and stewardship when integrated with conventional microbiological diagnostics.

## 1. Introduction

Infectious diseases remain a major cause of morbidity and mortality worldwide, representing a substantial burden for healthcare systems and highlighting the need for rapid and accurate diagnostic strategies to guide clinical decision-making [1,2]. For decades, conventional culture-based microbiological methods have been considered the diagnostic gold standard for pathogen identification and antimicrobial susceptibility testing. However, these approaches are intrinsically limited by prolonged turnaround times—often requiring 24 to 72 h—and reduced sensitivity, particularly in patients who have already received antimicrobial therapy [3,4]. These limitations are increasingly problematic in the context of the global rise of antimicrobial resistance (AMR), which undermines the effectiveness of empiric therapy and reinforces the urgency of timely pathogen identification combined with resistance profiling [5,6].

To overcome these challenges, syndromic molecular diagnostics have emerged as a transformative innovation in clinical microbiology. These platforms rely on multiplex nucleic acid amplification tests (NAATs) that enable the simultaneous detection of a wide range of bacterial, viral and fungal pathogens, together with clinically relevant resistance genes, directly from clinical specimens within a few hours [7,8,9]. By providing rapid and actionable results, syndromic panels support early targeted antimicrobial therapy, enhance antimicrobial stewardship programs and may reduce hospital length of stay, inappropriate antibiotic use and overall healthcare costs [10,11]. Among these technologies, the BioFire^®^ FilmArray^®^ system (BioMérieux, Marcy-l’Étoile, France) is one of the most widely adopted and extensively evaluated platforms in routine diagnostic workflows.

Bloodstream infections (BSIs) and sepsis exemplify clinical conditions in which diagnostic delays are associated with poor outcomes. Each hour of delay in the administration of effective antimicrobial therapy has been linked to increased mortality, particularly in patients with septic shock [12]. The BioFire^®^ FilmArray^®^ Blood Culture Identification 2 (BCID2) panel represents a significant advancement in this setting. This fully automated assay identifies 43 targets, including Gram-positive and Gram-negative bacteria, yeasts and ten key antimicrobial resistance genes, directly from positive blood cultures in approximately one hour [13]. While matrix-assisted laser desorption/ionization time-of-flight mass spectrometry (MALDI-TOF MS) has substantially reduced organism identification times, it does not routinely provide resistance gene detection. In contrast, molecular panels such as BCID2 integrate pathogen identification with resistance profiling, facilitating earlier escalation or de-escalation of therapy and improving antimicrobial stewardship in critically ill patients [10,14,15].

Lower respiratory tract infections (LRTIs), including community-acquired pneumonia (CAP), hospital-acquired pneumonia (HAP) and ventilator-associated pneumonia (VAP), remain among the leading causes of infection-related mortality worldwide [6,16]. Microbiological diagnosis in LRTIs is particularly challenging due to the presence of colonizing flora in respiratory samples and the rapid sterilization of cultures following antibiotic exposure [3]. The BioFire^®^ FilmArray^®^ Pneumonia (PN) Panel and PN Panel Plus have been developed to address these limitations. These assays provide semi-quantitative detection of 15 bacterial pathogens, atypical bacteria, respiratory viruses and multiple resistance markers directly from sputum, endotracheal aspirates and bronchoalveolar lavage (BAL) specimens [17,18]. The use of a logarithmic quantification scale (10^4^ to ≥10^7^ copies/mL) offers clinically relevant information that may assist clinicians in differentiating colonization from true infection, particularly in invasive lower respiratory tract samples [17,18,19].

Central nervous system infections (CNSIs), such as meningitis and encephalitis, represent medical emergencies associated with high morbidity and mortality and often present with non-specific clinical manifestations [20]. Conventional diagnostic methods based on culture or targeted PCR may be slow and insensitive, especially after the initiation of empiric antimicrobial therapy. The BioFire^®^ FilmArray^®^ Meningitis/Encephalitis (ME) Panel enables the simultaneous detection of 14 common bacterial, viral and fungal pathogens directly from cerebrospinal fluid (CSF) within approximately one hour [21]. Given that a substantial proportion of CNSIs are viral or self-limiting, rapid molecular diagnostics may help reduce unnecessary antimicrobial exposure and support more appropriate clinical management.

Similarly, gastrointestinal infections remain a frequent cause of illness in both community and hospitalized patients, yet conventional diagnostic techniques fail to identify an etiological agent in a significant proportion of cases. Syndromic molecular panels have demonstrated superior sensitivity and faster turnaround times compared with traditional culture-based and immunoassay methods, enabling earlier diagnosis and improved patient management [11].

Despite the clear diagnostic advantages of syndromic molecular testing, their real-world implementation requires careful evaluation. Issues such as diagnostic concordance with conventional methods, interpretation of polymicrobial results and cost-effectiveness within specific healthcare settings remain critical considerations [10]. Real-life data are therefore essential to define the microbiological performance and clinical impact of these technologies in routine practice.

The aim of this study was to evaluate the implementation and microbiological performance of multiple BioFire^®^ FilmArray^®^ syndromic panels within the routine diagnostic workflow of a tertiary-care hospital in central Italy. We analyzed the distribution of pathogens associated with bloodstream, lower respiratory tract, central nervous system and gastrointestinal infections in both hospitalized and community patients. In addition, we compared molecular diagnostic results with conventional culture-based methods to assess diagnostic agreement and to explore their potential implications for clinical management and antimicrobial stewardship.

## 2. Results

A total of 955 clinical specimens were analyzed using four BioFire^®^ FilmArray^®^ syndromic panels, including blood cultures (*n* = 400), lower respiratory tract samples *(n* = 309), CSF (*n* = 158) and stool specimens (*n* = 88). Overall, 698/955 samples (73.1%) tested positive for at least one microbial target, while 257/955 (26.9%) were negative across all panels.

**Figure 1:**
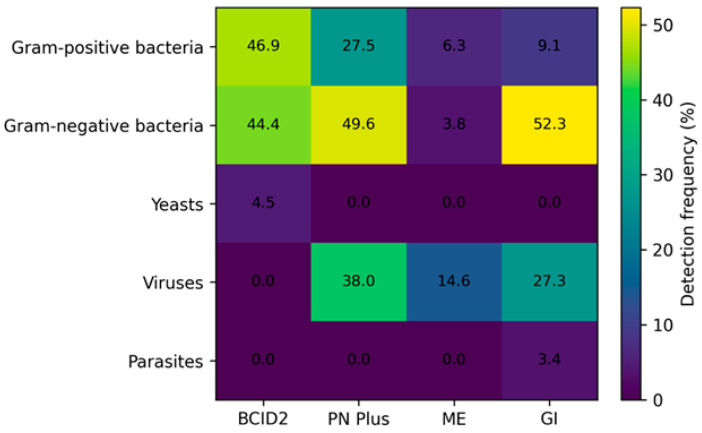
Heatmap showing the detection frequency (%) of microbial target categories across the four BioFire^®^ FilmArray^®^ panels evaluated in this study (BCID2, PN Plus, ME and GI). Values reported in each cell represent the percentage of positive detections relative to the total number of samples analyzed per panel. Color intensity and numerical annotations indicate increasing detection frequency.

The proportion of positive samples varied by clinical syndrome, ranging from 74.1% for the PN Plus Panel and 69.3% for the GI Panel, to 20.9% for the ME Panel. Blood cultures analyzed with the BCID2 panel showed the highest positivity rate (93.8%), reflecting the preselection of samples based on culture positivity. Figure 1 summarizes the distribution of detected microbial categories across panels, highlighting the predominance of Gram-negative bacteria in bloodstream and respiratory infections, viral pathogens in respiratory and CSF samples and a mixed bacterial–viral etiology in gastrointestinal infections.

Across all panels, monomicrobial detections predominated; however, polymicrobial results were frequently observed in respiratory and bloodstream samples, consistent with the complexity of these infection sites.

### 2.1. Bloodstream Infections: BCID2 Panel

Among the 400 positive blood cultures analyzed, 375 (93.8%) yielded at least one target detected by the BCID2 panel, while 25 samples (6.2%) were negative. Of these negative samples, 20 were culture-positive for organisms not included among BCID2 targets, whereas 5 were negative by both molecular and conventional methods.

A total of 440 microbial targets were detected, with monomicrobial infections accounting for 310/400 samples (77.5%), dual detections in 48/400 (12.0%) and polymicrobial (>2 targets) detections in 17/400 (4.3%). Gram-positive bacteria represented the most frequently detected group, followed by Gram-negative bacteria, while yeasts accounted for a smaller proportion (Figure 1).

Among Gram-negative organisms, *Escherichia coli* was the most prevalent pathogen (15.0%), followed by *Klebsiella pneumoniae* (8.7%), *Acinetobacter calcoaceticus–baumannii* complex (6.5%) and *Pseudomonas aeruginosa* (4.0%). Gram-positive detections were dominated by *Staphylococcus epidermidis* (19.3%), *Staphylococcus aureus* (16.7%) and other *Staphylococcus* species (10.8%), as well as *Enterococcus faecalis* (8.2%) and *E. faecium* (5.0%). Fungal pathogens were detected in 16/400 samples (4.0%), mainly *Candida albicans* and *Candida parapsilosis*.

Overall concordance with conventional culture was high, with a cumulative positive percent agreement (PPA) of 97.7% and negative percent agreement (NPA) of 99.8%. Discrepancies were limited and primarily involved polymicrobial samples (Table 1).

### 2.2. Lower Respiratory Tract Infections: Pneumonia Panel Plus

Of the 309 respiratory specimens analyzed, 229 (74.1%) were positive for at least one pathogen, while 80 (25.9%) were negative by the PN Plus Panel. A total of 408 targets were detected, reflecting frequent polymicrobial detections.

Monomicrobial results were observed in 110/309 samples (35.6%), while dual detections occurred in 78/309 (25.2%) and ≥3 targets in 44/309 (14.2%). Gram-negative bacteria represented the most frequent bacterial category, followed by Gram-positive bacteria, whereas viral pathogens were detected in 38.0% of samples (Figure 1).

Among bacterial pathogens, *Staphylococcus aureus* was the most frequently detected species (22.0%), followed by *Haemophilus influenzae* (13.3%), *Pseudomonas aeruginosa* (11.0%), *Klebsiella pneumoniae* (10.4%) and *A. calcoaceticus–baumannii* complex (7.8%). Viral detections were dominated by human rhinovirus/enterovirus (12.0%), seasonal coronaviruses (8.4%), influenza A virus (5.8%) and respiratory syncytial virus (3.2%).

Bacterial–viral co-detections were common, particularly in polymicrobial samples, underscoring the added value of multiplex testing in respiratory infections. Concordance with conventional culture was high for bacterial targets, with an overall PPA of 97.8% and NPA of 96.6%. Discordant results were mainly associated with culture-negative but molecular-positive samples (Table 2).

#### Correlation of Bacterial Loads

A total of 156 bacterial loads were analyzed. The comparison between reference culture and FilmArray^®^ showed a moderate positive correlation, with a coefficient of determination R^2^ = 0.31 (Pearson *r* = 0.56).

**Figure 2:**
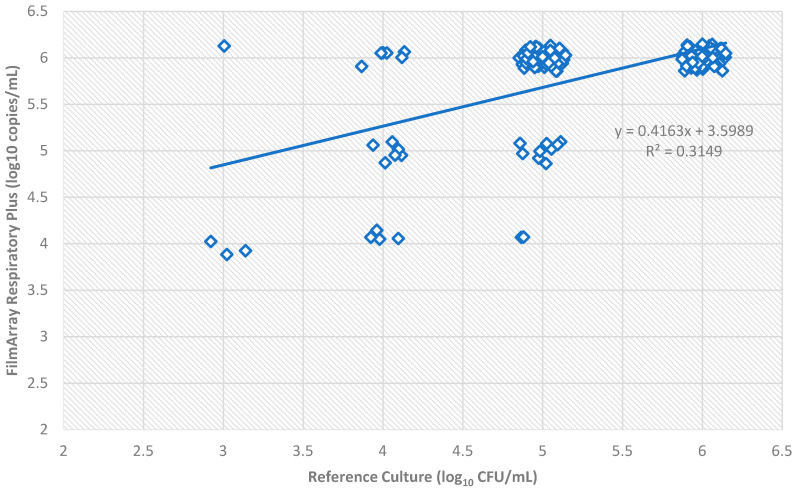
Scatter plot: correlation between traditional culture and the molecular semi-quantitative method for quantification of bacterial load in respiratory samples.

The scatter plot highlights a general trend where FilmArray^®^ values tend to be approximately 1 log_10_ higher than those obtained via culture (Figure 2). A significant cluster of samples was observed at the upper limit of quantification 10^6^. Despite the moderate R^2^ value, the association between the two methods remained statistically significant (*p* < 0.001), indicating that higher genomic loads detected by FilmArray^®^ generally correspond to higher viable bacterial counts in culture.

Exact Agreement was achieved in 108 out of 156 samples (69.2%). When a tolerance of ±1 log_10_ was applied (Essential Agreement), the concordance rate rose significantly to 97.4% (152/156 samples).

A systematic shift toward higher quantification was observed for the molecular method: in 28.8% of the total samples (45/156), FilmArray^®^ reported a bacterial load at least 1 log_10_ higher than culture. The most frequent discrepancy was observed in samples with 10^5^ CFU/mL in culture, of which 78.3% (36/46) were upgraded to ≥10^6^ copies/mL by FilmArray^®^. Only 3 samples (1.9%) showed a lower load in FilmArray^®^ compared to culture.

### 2.3. Central Nervous System Infections: ME Panel

Among the 158 CSF samples tested, 33 (20.9%) were positive for at least one target, while 125 (79.1%) were negative. Of the negative samples, 120 (96.0%) showed no growth by culture, whereas 5 (4.0%) yielded organisms not included in the ME panel targets.

Most positive samples were monomicrobial (31/158; 19.6%), with only 2 samples (1.3%) showing dual viral detections. Viral pathogens predominated, accounting for the majority of positive results (Figure 1). Enterovirus was the most frequently detected target (7.6%), followed by human herpesvirus 6 (3.8%) and varicella zoster virus (1.9%). Bacterial detections were less frequent and included *Streptococcus pneumoniae*, *Listeria monocytogenes* and *Haemophilus influenzae* (Table 3). All bacterial detections showed complete concordance with culture (PPA 100%), while viral detections lacked a conventional reference standard. The ME panel demonstrated a high overall NPA (99.6%).

### 2.4. Gastrointestinal Infections: GI Panel

The GI panel yielded positive results in 61/88 stool samples (69.3%), with 27 (30.7%) remaining negative. A total of 81 microbial targets were detected. Monomicrobial infections accounted for 44/88 samples (50.0%), while dual and polymicrobial detections occurred in 12/88 (13.6%) and 4/88 (4.5%) samples, respectively.

Bacterial pathogens were the most frequently detected category, followed by viral agents (Figure 1). *Campylobacter* spp. were the leading bacterial pathogens (23.9%), followed by Salmonella spp. (9.1%) and *Clostridioides difficile* producing toxin A/B (9.1%). Viral pathogens included norovirus GI/GII (15.9%), sapovirus (4.5%) and rotavirus A (3.4%). Parasitic infections were less frequent, with *Giardia lamblia* and *Cryptosporidium* detected in a limited number of samples.

Concordance with conventional methods was excellent for bacterial targets included in routine testing, with a cumulative PPA of 100% and an NPA of 96.9% (Table 4).

### 2.5. Detection of Antimicrobial Resistance Markers by BCID2 and Pneumonia Plus Panels

Antimicrobial resistance genes were detected in 170 clinical samples, including 119 positive blood cultures and 51 lower respiratory tract specimens. Resistance determinants were identified across multiple bacterial species and molecular results were compared with phenotypic antimicrobial susceptibility testing (AST) whenever the corresponding isolates were successfully recovered in culture (Table 5).

Among Gram-negative pathogens, the CTX-M extended-spectrum β-lactamase marker was detected in 35 isolates, including *Escherichia coli*, *Klebsiella pneumoniae* and *Proteus mirabilis*. In all cases, CTX-M detection showed 100% concordance with phenotypic resistance to third- and fourth-generation cephalosporins. Carbapenemase gene detection also demonstrated high diagnostic reliability. Specifically, the combined detection of NDM + OXA-48-like + CTX-M (*n* = 6), KPC + VIM (*n* = 3) and KPC + CTX-M (*n* = 6) was fully concordant (100%) with phenotypic results. Single carbapenemase markers, including KPC and IMP, were detected with complete concordance (100%) in both Enterobacterales and *Pseudomonas aeruginosa*. The VIM marker showed a slightly lower concordance, with phenotypic confirmation in 4 out of 5 isolates (80%). In all cases in which carbapenemase genes were detected by the molecular panels, enzyme production was independently confirmed using routine immunochromatographic assay.

Among Gram-positive pathogens, the detection of *vanA*/*vanB* genes in *Enterococcus faecium* isolates (*n* = 4) demonstrated 100% concordance with phenotypic resistance to vancomycin and/or teicoplanin. Finally, *mecA/C* and *MREJ* markers were identified in 97 staphylococcal isolates (*Staphylococcus aureus* and *Staphylococcus epidermidis*), with an overall concordance of 87.3% (76/87) with oxacillin resistance and/or cefoxitin screening positivity.

## 3. Discussion

The results of this study confirm the substantial clinical and diagnostic value of syndromic rapid molecular testing when integrated into routine clinical microbiology workflows. While conventional culture-based microbiology remains the reference standard for definitive pathogen isolation and phenotypic antimicrobial susceptibility testing, its intrinsic limitations—particularly prolonged turnaround time (TAT), reduced sensitivity in patients already receiving antimicrobial therapy and inability to detect fastidious or non-cultivable organisms—are well recognized [22,23]. In this context, syndromic molecular panels represent a paradigm shift, enabling rapid and comprehensive etiological characterization of infectious syndromes.

Across the different biological matrices analyzed, syndromic testing demonstrated a high diagnostic yield and excellent concordance with conventional culture, in line with previously published multicenter evaluations [24,25]. The observed variability in positivity rates among sample types reflects both the underlying epidemiology of infections and the different performance of culture-based diagnostics across anatomical sites. Importantly, molecular assays proved particularly effective in identifying polymicrobial infections and pathogens not readily detectable by routine methods, reinforcing their added value in complex clinical scenarios.

In bloodstream infections, the distribution of detected pathogens—dominated by Gram-positive cocci such as *Staphylococcus aureus* and coagulase-negative staphylococci, followed by Enterobacterales and non-fermenting Gram-negative bacteria—mirrors the epidemiology reported in hospital- and community-acquired bacteremia [26,27]. The ability of the molecular panel to simultaneously detect multiple organisms is of particular relevance in polymicrobial sepsis, a condition in which conventional identification methods, including MALDI-TOF MS applied directly from positive blood culture bottles, may fail to identify all causative agents [23,28].

A major strength of syndromic molecular diagnostics is the simultaneous detection of key antimicrobial resistance genes, allowing the deduction of resistance or susceptibility profiles for clinically relevant antibiotic classes. In this study, the identification of determinants such as *mecA/C*, *vanA/B*, *blaKPC*, *blaNDM*, *blaVIM*, *blaIMP*, *blaOXA-48-like* and *blaCTX-M* enabled early prediction of resistance to β-lactams, carbapenems and glycopeptides, well in advance of phenotypic susceptibility testing. This “molecular antibiogram” provides actionable information to guide early antimicrobial optimization, particularly in critically ill patients [14,15,16].

From a therapeutic perspective, early recognition of carbapenemase genes supports the timely use of novel β-lactam/β-lactamase inhibitor combinations (BLICs), such as ceftazidime/avibactam, meropenem/vaborbactam and imipenem/relebactam for KPC-producing Enterobacterales, while also identifying scenarios in which these agents may be ineffective, such as infections caused by metallo-β-lactamase producers, for which cefiderocol and aztreonam/avibactam represent valuable options [23,29]. Several studies have demonstrated that early, targeted therapy based on rapid molecular results is associated with improved clinical outcomes and reduced mortality in severe Gram-negative infections [10,14,15,30].

Discordant results between molecular assays and culture were limited and mainly involved Gram-positive cocci, particularly staphylococci carrying mecA. These discrepancies may be explained by the high analytical sensitivity of molecular methods, which can detect low bacterial loads or residual DNA from non-viable organisms. This may reflect the presence of low-level contamination by coagulase-negative staphylococci not recovered by culture or not reported due to their interpretation as contaminants. Alternatively, mixed populations with a minor resistant subpopulation may be detected at the molecular level but not phenotypically expressed in culture [31]. Given the retrospective nature of the analysis, it was not possible to investigate the discrepancies for *mecA* detection on a case-by-case basis; however, evidence from the literature supports the explanations above. These findings highlight the need for careful clinical and microbiological interpretation of molecular resistance markers, especially for *mecA* detection in staphylococci, and reinforce the importance of correlating molecular results with culture, Gram stain, and clinical context [14,15,16,31].

Beyond bloodstream infections, the application of syndromic panels to respiratory, gastrointestinal and cerebrospinal fluid samples provided additional diagnostic benefits. The detection of viral pathogens and atypical bacteria not identifiable by routine culture supports more accurate etiological diagnosis and contributes to antimicrobial stewardship by reducing unnecessary antibiotic use [32,33]. In central nervous system infections, rapid identification of viral etiologies enables earlier discontinuation of empiric antibacterial therapy, with clear benefits in terms of toxicity reduction and resource optimization [21,34].

The analysis of lower respiratory tract specimens using the FilmArray^®^ Pneumonia Plus panel demonstrated a high positivity rate (74.1%) and frequent polymicrobial detections, highlighting the panel’s utility in complex respiratory infections. Concordance with conventional culture for bacterial targets was high (PPA 97.8%, NPA 96.6%), supporting its reliability for routine diagnostics. The semi-quantitative assessment of bacterial loads revealed a moderate correlation with culture-based counts (R^2^ = 0.282), with a systematic tendency for FilmArray^®^ to report approximately 1 log_10_ higher genomic copies compared to culture. This shift likely reflects differences in the detection of viable versus total nucleic acid, particularly in polymicrobial or low-load samples. Despite the moderate R^2^, essential agreement (±1 log_10_) reached 97.4%, confirming that semi-quantitative results are broadly consistent with culture and can provide useful information for pathogen burden estimation, while acknowledging that absolute bacterial loads may be overestimated [17,35,36].

One of the most clinically relevant advantages of syndromic molecular diagnostics is the marked reduction in TAT. While culture-based identification and susceptibility testing typically require 24–72 h, syndromic panels provide results within approximately one hour. Numerous studies have demonstrated that this reduction translates into earlier administration of appropriate antimicrobial therapy, reduced length of hospital stay and improved patient outcomes, particularly in sepsis and septic shock, where delays in effective treatment are directly associated with increased mortality [14,15,22,37].

Despite these advantages, molecular diagnostics have inherent limitations. They do not provide phenotypic antimicrobial susceptibility testing, cannot detect resistance mechanisms outside the targeted gene panel and do not replace the need for organism isolation for epidemiological surveillance, infection control and further characterization. Additionally, the higher cost of syndromic panels necessitates their judicious use within well-defined diagnostic algorithms.

For these reasons, syndromic molecular assays should be viewed not as a replacement but as a complementary tool to conventional microbiology. An integrated diagnostic strategy combining rapid molecular testing, traditional culture, MALDI-TOF MS and structured antimicrobial stewardship programs represents the most effective approach to maximize diagnostic accuracy, optimize antimicrobial therapy and limit the spread of antimicrobial resistance.

This study has several strengths. A key strength is the simultaneous implementation of four different syndromic panels within the routine diagnostic workflow over an extensive period of more than three years. Furthermore, the large cohort of clinical specimens, combined with a rigorous correlation analysis between semi-quantitative genomic bacterial loads and viable culture counts in respiratory samples, provides real-world evidence of the diagnostic reliability of these molecular platforms. In addition, the panels targeting resistance genes were evaluated in a real-world epidemiological setting where infections caused by multidrug-resistant pathogens—such as carbapenemase- or ESBL-producing Enterobacterales, vancomycin-resistant enterococci, and methicillin-resistant *Staphylococcus* spp.—are endemic, highlighting their applicability and reliability in a high-MDR prevalence context.

However, several limitations should be considered. First, the study is retrospective, and the positivity rate for certain specific pathogens was either low or absent. Second, no gold-standard reference methods were available for direct performance comparison of non-cultivable organisms, including viruses, fastidious bacteria, and parasites. Furthermore, this evaluation focused primarily on microbiological performance and did not assess the actual clinical impact of the molecular results on antimicrobial stewardship interventions or patient outcomes. Consequently, further prospective studies are necessary to evaluate the clinical impact and the cost-effectiveness of implementing BioFire^®^ FilmArray^®^ panels in routine diagnostic workflows.

## 4. Materials and Methods

### 4.1. Study Design and Clinical Specimen Collection

This retrospective study was conducted at the Microbiology Unit of a secondary-care hospital in Central Italy and covered the period from January 2022 to June 2025. The study aimed to evaluate the integration and microbiological performance of the BioFire^®^ FilmArray^®^ syndromic multiplex PCR system within a routine clinical microbiology workflow. A total of 955 clinical specimens were collected from both hospitalized patients—admitted to intensive care units, emergency departments, and medical or surgical wards—and community-based patients. FilmArray testing was requested by the treating clinicians based on clinical suspicion and urgency, and samples were selected according to the suspected infectious syndrome and categorized as follows: 400 positive blood cultures for suspected bloodstream infections; 309 lower respiratory tract specimens, including bronchoalveolar lavage (BAL), endotracheal aspirates (ETAs), and bronchial aspirates, for pneumonia; 158 cerebrospinal fluid (CSF) samples for suspected meningitis or encephalitis; and 88 stool specimens for gastrointestinal infections. All samples were processed immediately upon arrival in the laboratory or, in the case of blood cultures, immediately after a positive signal from automated culture systems, in order to minimize diagnostic turnaround time.

### 4.2. Syndromic Molecular Testing: BioFire^®^ FilmArray^®^ System

Molecular testing was performed using the BioFire^®^ FilmArray^®^ platform (bioMérieux, Marcy l’Etoile, France). Briefly, the BioFire^®^ FilmArray^®^ system is a fully automated, multiplex PCR-based platform that integrates nucleic acid extraction, nested PCR amplification, and melt-curve analysis within a closed, single-use pouch. The system differentiates pathogens by using target-specific primers for each microorganism, allowing simultaneous detection and identification of multiple bacterial, viral, and fungal pathogens directly from clinical specimens. All assays were performed strictly according to the manufacturer’s instructions (https://www.biomerieux.com). Four different FilmArray^®^ panels were used depending on specimen type and clinical indication: Blood Culture Identification 2 (BCID2), Pneumonia Plus (PN Plus), Meningitis/Encephalitis (ME) and Gastrointestinal (GI) panels. A detailed overview of each panel and its detected targets is provided in Table 6. For respiratory samples, the PN Plus Panel provided semi-quantitative results expressed as logarithmic bins ranging from 10^4^ to ≥10^7^ genomic copies/mL.

### 4.3. Conventional Culture-Based Diagnostics and Laboratory Automation

In parallel with molecular testing, all specimens were processed using conventional microbiological methods, which served as the reference standard for performance comparison.

Blood cultures were incubated and monitored using the Bact/Alert^®^ Virtuo system (bioMérieux). Upon positivity, aliquots were immediately subjected to Gram staining and BCID2 testing, while subcultures were prepared on solid media.

Respiratory, gastrointestinal and blood culture samples were processed using the WASP-LAB^®^ platform (COPAN Italia, Brescia, Italy) for automated specimen inoculation and streaking. Samples were plated on selective and enriched media according to specimen type and clinical suspicion. Incubation was performed under aerobic or CO_2_-enriched conditions, as appropriate for the target pathogens.

### 4.4. Pathogen Identification and Antimicrobial Susceptibility Testing

Microbial identification from solid media was performed using MALDI-TOF mass spectrometry (VITEK MS, bioMérieux). Single colonies were applied to target slides, overlaid with matrix solution and analyzed by comparison with the manufacturer’s reference database. Antimicrobial susceptibility testing (AST) was primarily conducted using the VITEK 2 automated system (bioMérieux), which determines minimum inhibitory concentrations (MICs) through kinetic fluorescence analysis. All AST results were interpreted according to the current European Committee on Antimicrobial Susceptibility Testing (EUCAST) clinical breakpoints. In cases where carbapenemase production was suspected, the CARBA 5 immunochromatographic test (NG Biotech, Guipry, France) was performed for the phenotypic confirmation of carbapenemase enzyme production.

### 4.5. Performance Analysis and Statistical Methods

Comparative analysis between FilmArray results and conventional culture focused on pathogen detection frequency, diagnostic concordance and semi-quantitative agreement. Detection frequency was expressed as the percentage of positive samples for each pathogen. Diagnostic performance was assessed using Positive Percent Agreement (PPA) and Negative Percent Agreement (NPA), defined as the proportion of culture-positive and culture-negative samples correctly identified by the molecular assays, respectively.

For respiratory samples, semi-quantitative molecular results obtained with the PN Plus Panel (log_10_ copies/mL) were compared with quantitative culture results (log_10_ CFU/mL). Values ≥ 10^6^ were capped at 10^6^ for analysis. Data were log-transformed to approximate normal distribution. Correlation was assessed using Pearson’s correlation coefficient (r) and coefficient of determination (R^2^). Exact Agreement (EA) was defined as identical log_10_ values, while Essential Agreement (ESA) was defined as a difference of ≤1 log_10_ unit. Discrepancies were classified as positive or negative bias depending on the direction of deviation. Statistical analyses were performed using Microsoft Excel and GraphPad Prism (GraphPad Software, Boston, MA, USA; https://www.graphpad.com). A *p*-value ≤ 0.05 was considered statistically significant.

For antimicrobial resistance gene detection, the correlation between resistance markers identified by the FilmArray^®^ panels and the corresponding phenotypic resistance observed by AST was assessed, along with concordance with routine carbapenemase detection using the immunochromatographic assay (NG-test Carba 5; NG Biotech, Guipry, France).

## 5. Conclusions

This study demonstrates that syndromic molecular diagnostics provide significant added value to routine microbiological testing by delivering rapid, comprehensive and clinically actionable information. The ability to identify pathogens and key resistance determinants within a markedly reduced timeframe supports earlier therapeutic optimization, improved antimicrobial stewardship and better clinical outcomes, particularly in severe and time-critical infections.

However, the limitations of molecular testing underscore the necessity of maintaining conventional culture-based diagnostics for phenotypic susceptibility testing and comprehensive microbiological evaluation. The optimal diagnostic approach lies in the integration of syndromic molecular panels with traditional methods within a multidisciplinary framework. Such an integrated strategy allows laboratories and clinicians to fully exploit the strengths of each methodology, ensuring high-quality patient care while addressing the ongoing challenge of antimicrobial resistance.

## Figures and Tables

**Table 1:** BioFire^®^ FilmArray^®^ BCID2 Panel results: distribution of detected microbial targets and concordance with conventional culture-based results.

Pathogen Target	No. Detection (%)	1 Target	2 Targets	>2 Targets	Positive Agreement (%)	Negative Agreement (%)
Bacteria						
*E. coli*	60/400 (15)	51	6	3	60/63 (95.2)	337/337 (100)
*E. cloacae complex*	9/400 (2.3)	9			9/9 (100)	391/391 (100)
*K. oxytoca*	7/400 (1.8)	4	2	1	6/7 (85.7)	392/393 (99.7)
*K. pneumoniae*	35/400 (8.7)	27	5	3	34/34 (100)	365/366 (99.7)
*P. aeruginosa*	16/400 (4)	10	4	2	13/14 (92.9)	383/386 (99.2)
*S. marcescens*	4/400 (1)	2	1	1	3/3 (100)	396/397 (99.7)
*Proteus* spp.	11/400 (2.8)	8	1	2	11/11 (100)	389/389 (100)
*A. baumannii* cpx	26/400 (6.5)	10	9	7	23/25 (92)	372/375 (99.2)
*Enterobacterales* ^a^	4/400 (1)	4			4/4 (100)	396/396 (100)
*S. maltophilia*	2/400 (0.5)	2			2/2 (100)	398/398 (100)
*K. aerogenes*	1/400 (0.3)	1			1/1 (100)	399/399 (100)
*Salmonella* spp.	0/400				NA	400/400 (100)
*B. fragilis*	2/400 (0.5)	2			2/2 (100)	398/398 (100)
*N. meningitidis*	0/400				NA	400/400 (100)
*S. aureus*	67/400 (16.7)	59	7	1	67/67 (100)	347/347 (100)
*S. epidermidis*	77/400 (19.3)	49	20	8	70/70 (100)	330/337 (97.9)
*S. lugdunensis*	3/400 (0.8)	1	2		2/2 (100)	397/398 (99.7)
*Staphylococcus* spp. ^b^	43/400 (10.8)	30	10	3	42/43 (97.7)	356/357 (99.7)
*E. faecalis*	33/400 (8.2)	15	11	7	31/32 (96.9)	366/368 (99.4)
*E. faecium*	20/400 (5)	7	7	6	19/19 (100)	382/383 (99.7)
*S. pneumoniae*	3/400 (0.8)	2		1	2/2 (100)	397/398 (99.7)
*S. pyogenes*	1/400 (0.3)	1			1/1 (100)	399/399 (100)
*Streptococcus* spp.	15/400 (3.8)	7	6	2	13/13 (100)	385/387 (99.5)
*S. agalactiae*	1/400 (0.3)	1			1/1 (100)	399/399 (100)
*L. monocytogenes*	1/400 (0.3)	1			1/1 (100)	399/399 (100)
Yeasts						
*C. albicans*	7/400 (1.7)	4	1	2	5/6 (83.3)	392/394 (99.5)
*C. glabrata*	3/400 (0.8)		2	1	2/2 (100)	397/398 (99.7)
*Candida tropicalis*	2/400 (0.5)	2			2/2 (100)	398/398 (100)
*C. parapsilosis*	4/400 (1)	1	2	1	4/4 (100)	396/396 (100)
*C. auris*	0/400				NA	400/400 (100)
*C. krusei*	0/400				NA	400/400 (100)
*Cryptococcus neoformans/gattii*	0/400				NA	400/400 (100)
Total samples (*n* = 400)	375 (93.75)	310/400 (77.5)	48/400 (12)	17/400 (42.5)	430/440 (97.7)	11,997/12,024 (99.8)

^a^ Only samples positive for Enterobacterales species other than those specifically targeted by the panel were included in the analysis. ^b^ Only samples positive for *Staphylococcus* species other than those specifically targeted by the panel were included in the analysis. Abbreviation: NA, not applicable.

**Table 2:** BioFire^®^ FilmArray^®^ Pneumonia Panel Plus results: distribution of detected microbial targets and concordance with conventional culture-based results.

Pathogen Target	Number of Detection (%)	1 Target	2 Targets	>2 Targets	Positive Agreement (%)	Negative Agreement (%)
Bacteria						
*E. coli*	23/309 (7.4)	4	8	11	10/10 (100)	286/299 (95.6)
*S. aureus*	68/309 (22)	19	22	27	25/26 (96.1)	240/283 (84.8)
*H. influenzae*	41/309 (13.3)	13	18	10	17/17 (100)	268/292 (91.8)
*Proteus* spp.	12/309 (3.9)	1	4	7	7/7 (100)	297/302 (98.3)
*M. catarrhalis*	12/309 (3.9)	1	6	5	1/1 (100)	297/308 (96.4)
*S. pneumoniae*	15/309 (4.9)	4	4	7	1/1 (100)	294/308 (95.5)
*A. baumannii* cpx	24/309 (7.8)	10	8	6	18/19 (94.7)	284/290 (97.9)
*P. aeruginosa*	34/309 (11)	10	12	12	24/24 (100)	275/285 (96.5)
*E. cloacae* cpx	9/309 (2.9)		5	4	5/5 (100)	300/304 (98.7)
*K. pneumoniae*	32/309 (10.4)	10	11	11	18/20 (90)	275/289 (95.1)
*K. oxytoca*	5/309 (1.6)		3	2	3/3 (100)	304/306 (99.4)
*K. aerogenes*	0/309				NA	309/309 (100)
*S. marcescens*	6/309 (1.9)	2	2	2	2/2 (100)	303/307 (98.7)
*S. agalactiae*	0/309				NA	309/309 (100)
*S. pyogenes*	2/309 (0.6)	1	1		1/1 (100)	307/308 (99.7)
*C. pneumoniae*	0/309				NA	NA
*L. pneumophila*	3/309 (1)	1	2		NA	NA
*M. pneumoniae*	4/309 (1.3)	4			NA	NA
Viruses						
*Adenovirus*	6/309 (1.9)		3	3	NA	NA
*Coronavirus*	26/309 (8.4)	3	12	11	NA	NA
*Metapneumovirus*	10/309 (3.2%)	5	1	4	NA	NA
*Rhinovirus/Enterovirus*	37/309 (12)	8	17	12	NA	NA
*Influenza A Virus*	18/309 (5.8)	4	10	4	NA	NA
*Influenza B Virus*	2/309 (0.6)	2			NA	NA
*Parainfluenza Virus*	9/309 (2.9)	5	2	2	NA	NA
*Respiratory Syncytial Virus*	10/309 (3.2)	3	5	2	NA	NA
Total samples (*n* = 309)	408	110/309 (35.6)	78/309 (25)	44/309 (14.2)	132/135 (97.8)	4348/4499 (96.6)

Abbreviation: cpx, complex; NA, not applicable.

**Table 3:** BioFire^®^ FilmArray^®^ ME Panel results: distribution of detected microbial targets and concordance with conventional culture-based results.

Pathogen Target	Number of Detection (%)	1 Target	2 Targets	Positive Agreement (%)	Negative Agreement (%)
Bacteria					
*S. agalactiae*	1/158 (0.6)	1		1/1 (100)	157/157 (100)
*S. pneumoniae*	5/158 (3.2)	5		3/3 (100)	153/155 (98.7)
*L. monocytogenes*	3/158 (1.9)	3		2/2 (100)	155/156 (99.4)
*H. influenzae*	2/158 (1.3)	2		1/1 (100)	156/157 (99.4)
*N. meningitidis*	1/158 (0.6)	1		1/1 (100)	157/157 (100)
*E. coli K1*	0/158			NA	158/158 (100)
Viruses					
*Enterovirus*	12/158 (7.6)	12		NA	NA
*Cytomegalovirus*	1/158 (0.6)		1	NA	NA
*Herpesvirus 6*	6/158 (3.8)	4	2	NA	NA
*Varicella zoster virus*	3/158 (1.9)	3		NA	NA
*Human parechovirus*	1/158 (0.6)		1	NA	NA
*Herpes simplex virus 1*	0/158			NA	NA
*Herpes simplex virus 2*	0/158			NA	NA
Yeasts					
*Cyptococcus neoformans/gatti*	0/158			NA	158/158
Total samples (*n* = 158)	35	31/158 (19.6)	2/158 (1.3)	8/8 (100)	1094/1098 (99.6)

Abbreviation: NA, not applicable.

**Table 4:** BioFire^®^ FilmArray^®^ GI Panel results: distribution of detected microbial targets and concordance with conventional culture-based results.

Pathogen Target	Number of Detection (%)	1 Target	2 Targets	>2 Targets	Positive Agreement (%)	Negative Agreement (%)
Bacteria						
*C. difficile toxin A/B ^a^*	8/88 (9.1)	5	2	1	6/6 (100)	80/82 (97.6)
*Campylobacter*	21/88 (23.9)	20	1		16/16 (100)	67/72 (93)
*Salmonella*	8/88 (9.1)	3	5		7/7 (100)	80/81 (98.8)
*Y. enterocolitica*	3/88 (3.4)	1	1	1	1/1 (100)	85/87 (97.7)
*Vibrio ^b^*	0/88				NA	NA
*P. shigelloides*	0/88				NA	NA
*EAEC*	5/88 (5.7)		1	4	NA	NA
*EPEC*	8/88 (9.1)	3	4	1	NA	NA
*ETEC*	0/88				NA	NA
*STEC*	0/88				NA	NA
*E. coli O157*	0/88				NA	NA
*Shigella/E. coli enteroinvasivo (EIEC)*	1/88 (1.1)			1	NA	NA
Viruses						
*Adenovirus F 40/41*	3/88 (3.4)	1	2		NA	NA
*Astrovirus*	0/88				NA	NA
*Norovirus GI/GII*	14/88 (15.9)	10	3	1	NA	NA
*Rotavirus A*	3/88 (3.4)	1	1	1	NA	NA
Parasites						
*Sapovirus (GI, II, IV, e V)*	4/88 (4.5)		3	1	NA	NA
*Cryptosporidium*	1/88 (1.1)			1	NA	NA
*Cyclospora cayetanensis*	0/88				NA	NA
*Entamoeba histolytica*	0/88				NA	NA
*Giardia lamblia*	2/88 (2.3)		1	1	NA	NA
Total samples (*n* = 88)	81	44 (50)	12 (13.6)	4 (4.5)	30/30 (100)	312/322 (96.9)

^a^ The STANDARD™ F *C. difficile* Toxin A/B FIA (SD Biosensor, Inc., Suwon-si, Gyeonggi-do, Republic of Korea) was utilized as the conventional routine method for concordance analysis. ^b^ *V. parahaemolyticus*/*V. vulnificus*/*V. cholerae* Abbreviations: NA, not applicable; EAEC, Enteroaggregative *E. coli*; EPEC, Enteropathogenic *E. coli*, ETEC; Enterotoxigenic *E. coli*; STEC, Shiga toxin-producing *E. coli.*

**Table 5:** Resistance markers detected by BCID2 and Pneumonia Plus panels and correlation with conventional phenotypic results.

FilmArray Results		Agreement with Phenotypic Results ^a^	
Resistance Marker	Bacterial Species	Expected Phenotype ^b^	
CTX-M	*E.coli* (*n* = 24) *K. pneumoniae* (*n* = 8) *P. mirabilis* (*n* = 3)	Resistance to 3rd and 4th generation cephalosporins (ceftazidime, cefotaxime) or MIC > 1 mg/L.	33/33 (100%)
NDM + OXA-48like + CTX-M	*K. pneumoniae* (*n* = 6)	Resistance to carbapenems (meropenem, ertapenem) or MIC > 0.12 mg/L. Resistance or elevated MICs for new BL/BLI combinations (e.g., CAZ/AVI, MEM/VAB, IMP/REL).	6/6 (100%)
OXA-48like + CTX-M	*K. pneumoniae* (*n* = 2)	Resistance to carbapenems (meropenem, ertapenem) or MIC > 0.12 mg/L.	NA
KPC + CTX-M	*E.coli* (*n* = 1) *K. pneumoniae* (*n* = 5)	Resistance to carbapenems (meropenem, ertapenem) or MIC > 0.12 mg/L.	4/4 (100%)
KPC + VIM	*K. pneumoniae* (*n* = 3)	Resistance to carbapenems (meropenem, ertapenem) or MIC > 0.12 mg/L. Resistance or elevated MICs for new BL/BLI combinations.	3/3 (100%)
KPC	*K. pneumoniae* (*n* = 14) *E.coli* (*n* = 1) *Proteus* spp. (*n* = 1)	Resistance to carbapenems (meropenem, ertapenem) or MIC > 0.12 mg/L.	10/10 (100%)
IMP	*P. aeruginosa* (*n* = 1)	Resistance to carbapenems (meropenem, ertapenem). Resistance or elevated MICs for new BL/BLI combinations.	1/1 (100%)
VIM	*P. aeruginosa* (*n* = 5) *Proteus* spp. (*n* = 1) *S. marcescens* (*n* = 1)	Resistance to carbapenems (meropenem, ertapenem) or MIC > 0.12 mg/L (for Enterobacterales). Resistance or elevated MICs for new BL/BLI combinations.	4/5 (80%)
*vanA/B*	*Enterococcus faecium* (*n* = 4)	Resistance to vancomycin and/or teicoplanin.	4/4 (100%)
*mecA/C* and *MREJ*	*S. aureus* (*n* = 29) *S. epidermidis* (*n* = 68)	Resistance to oxacillin and/or positivity to the cefoxitin screen.	76/87 (87.3%)

^a^ Concordance with phenotypic results was evaluated if the bacteria were isolated in culture. ^b^ According to current EUCAST guidelines. Abbreviations: BL/BLI, β-lactam/β-lactamase inhibitor; CAZ/AVI, ceftazidime/avibactam; MEM/VAB, meropenem/vaborbactam; IMP/REL, imipenem/relebactam. Abbreviation: NA, not applicable (bacteria not recovered in culture).

**Table 6:** BioFire^®^ FilmArray^®^ panels used in this study and their respective targets.

FILMARRAY Panel	Sample Type	Bacterial Targets	Viral Targets	Fungal/Parasitic Targets	Antimicrobial Resistance Genes
BCID2	Positive blood cultures	11 Gram-positive, 15 Gram-negative	–	7 yeasts	*mecA/C*, *MREJ*, *vanA/B*, *blaKPC*, *blaNDM*, *blaVIM*, *blaOXA-48-like*, *blaIMP*, *blaCTX-M*
PN Plus ^a^	Respiratory samples (BAL, ETA, sputum)	15 (semi-quantitative) + 3 atypical	9 + MERS-CoV	–	*mecA/C*, *MREJ*, *blaCTX-M*, *blaKPC*, *blaNDM*, *blaVIM*, *blaOXA-48-like*
ME	CSF	6	7	*Cryptococcus neoformans*/*gattii*	–
GI	Stool	13	5	4 parasites	–

^a^ The BioFire^®^ FilmArray^®^ Pneumonia Plus Panel includes all targets of the standard Pneumonia Panel with the addition of Middle East Respiratory Syndrome Coronavirus (MERS-CoV). PN Plus provides semi-quantitative bacterial results that are reported as log_10_ genomic copies/mL, ranging from 10^4^ to ≥10^7^.

## Data Availability

The datasets generated and analyzed during the current study are available from the corresponding author upon reasonable request.
